# Supplementary material for: Remote sensing of emperor penguin abundance and breeding success
Source: Nat Commun. 2024 May 29;15:4419. doi: 10.1038/s41467-024-48239-8 (PMC11137044; doi:10.1038/s41467-024-48239-8)
Supplement: Supplementary file 1 — Supplementary Information [file 41467_2024_48239_MOESM1_ESM.pdf]

Remote sensing of emperor penguin abundance and breeding success

Alexander Winterl[1], Sebastian Richter[1], Aymeric Houstin[1,2,3], Téo Barracho[3,4,5], Matthieu Boureau[3], Clément Cornec[3,6], Douglas Couet[3], Robin Cristofari[3,7], Claire Eiselt[3], Ben Fabry[1], Adélie Krellenstein[3], Christoph Mark[1], Astrid Mainka[1], Delphine Ménard[3], Jennifer Morinay[3], Susie Pottier[3], Elodie Schloesing[3], Céline Le Bohec[3,4,8^] and Daniel P. Zitterbart[1,2^]

[1] Department of Physics, Friedrich-Alexander Universität Erlangen-Nürnberg, Erlangen, Germany.
[2] Department of Applied Ocean Physics and Engineering, Woods Hole Oceanographic Institution, Woods Hole, USA.
[3] Université de Strasbourg, CNRS, IPHC UMR 7178, F-67000 Strasbourg, France.
[4] CEFE, Université de Montpellier, CNRS, EPHE, IRD, Montpellier, France.
[5] University of Moncton, Canada Research Chair in Polar and Boreal Ecology and Centre d'Études Nordiques, Department of Biology, Moncton, New Brunswick, Canada.
[6] ENES Bioacoustics Research Laboratory, CRNL, CNRS, Inserm, University of Saint-Etienne, Saint-Etienne, France.
[7] Institute of Biotechnology, University of Helsinki, Helsinki, Finland.
[8] Centre Scientifique de Monaco, Département de Biologie Polaire, Monaco, Principality of Monaco.

^ These authors jointly supervised this work.

Corresponding authors:
Alexander Winterl (alexander.winterl@fau.de)
Daniel P. Zitterbart (dpz@whoi.edu)

Supplement

Supplementary Note 1

We estimated the values for the phenological model parameters using Bayesian inference based on Markov Chain Monte Carlo (MCMC) sampling implemented in Python [44,45]. The prior distributions for all parameters were uniform in between the boundaries given in Table 1. The counted number of individuals is subject to a statistical measurement error and accordingly follows a distribution. We assumed that the main counting error is relative: the human observers miscount by a ratio rather than a fixed number. Consequently and in accordance with literature [23], we chose the log-normal distribution as the individual counts error distribution, because its width scales approximately linear with the mean value. We tackled numerical issues at very low numbers (<100 individuals) by adding a constant offset number of non-breeding individuals that could be present regardless of the phenology. The width of the log-normal

distribution and the offset number are free parameters. We chose an exponential distribution as the prior for both parameters to ensure minimization of error distribution width during the sampling process. Table 1 contains all free parameters of the model, the numerical range, prior distribution, and a brief description.

We used the 511 counts of adult individuals to optimize the parameters of our model. We sampled the 208 parameters (16 per season, 10 seasons at Point Géologie, 3 seasons at Atka Bay) of the model with the No-U-Turn-Sampler [45] for 200 tuning and 200 draw iterations in 8 independent chains. The parameters and therefore sampling statistics for different seasons and colonies are independent by model definition. The tuning samples were discarded after the sampling process. We use the pooled 1600 draw samples to compute means and errors for all parameters and other result plots. We computed a R-hat statistics [46] of less than 1.02 for each of the parameters. Consequently, the sampling has converged. Sup. Fig 2 shows a trace plot containing the trace and kernel density estimate for all parameters.

We used MCMC sampling and Bayesian statistics to infer the parameters of the windchill model while only using the measured areas and meteorological data as inputs. We applied non-informative normal distributions as priors for the linear factors and the transition temperature ($c_T = 1 / b_0$, c_W, c_R, c_H, T_c). Again, we modeled the error distribution of the density (ρ) as a log-normal distribution with standard deviation σ. Analogous to the phenological model, we choose an exponential distribution as the prior for σ.

Supplementary Figure 1

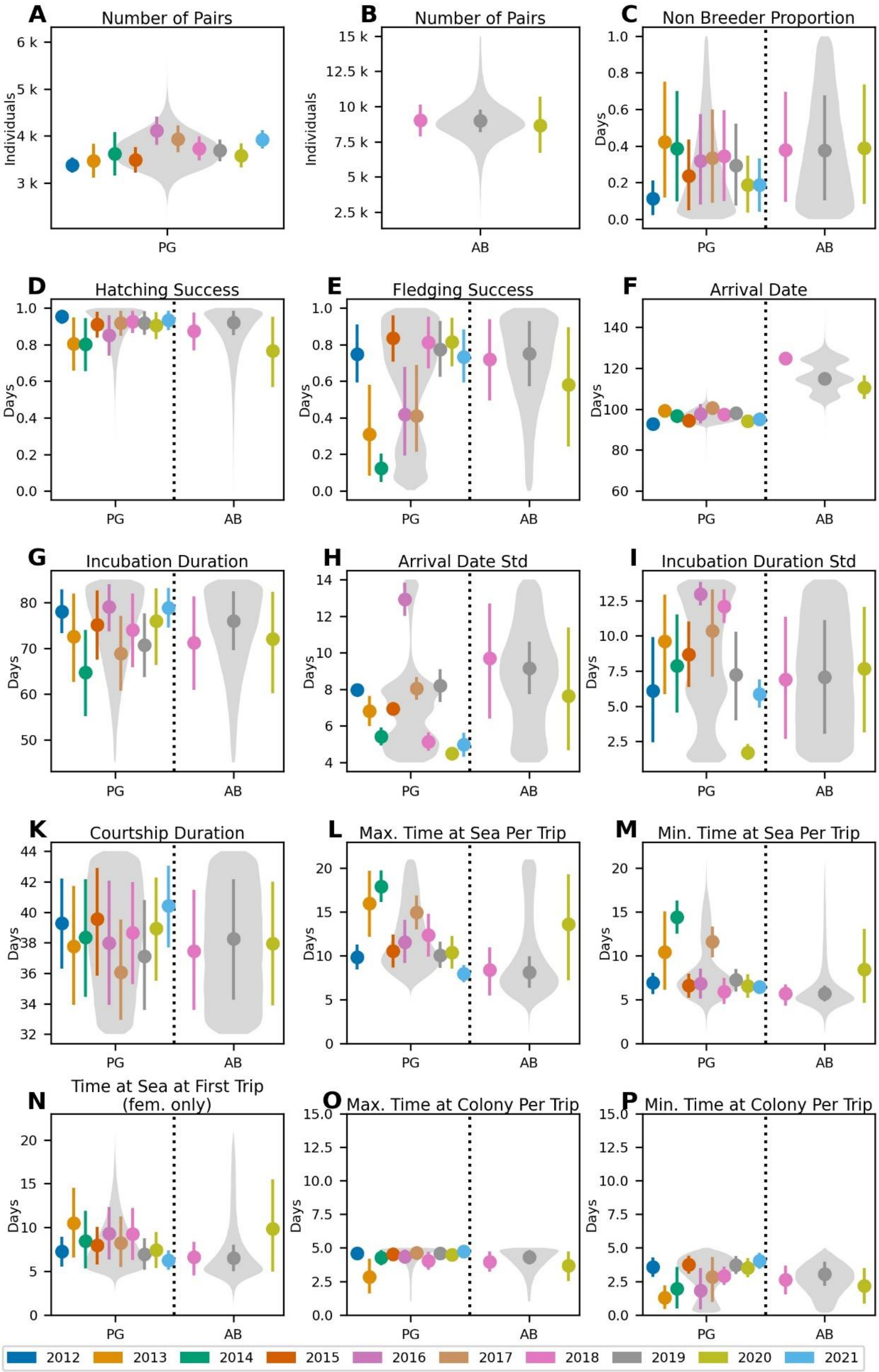

Sup. Fig. 1. Distribution plots of the model parameters for Pointe Géologie and Atka

Bay colonies. In each panel, the left part shows the parameters for Pointe Géologie colony (PG) and the right part for Atka Bay colony (AB). The points show the parameter value for each year, while the violin plots show the kernel density estimation for the parameter.

Supplementary Table 1

This table contains the numerical values of the best estimate for the parameters of the phenological model.

Colony	Season	Number of Pairs	Non Breeder Proportion	Hatching Success	Fledging Success	Arrival Date	Arrival Date Std	Courtship Duration	Female Absence Duration	Female Absence Duration Std	Max. Time at Colony Per Trip	Min. Time at Colony Per Trip	Max. Time at Sea Per Trip	Time at Sea at First Trip (fem. only)	Min. Time at Sea Per Trip	Relative Error	Offset Error
**Unit**		1	1	1	1	d	d	d	d	d	d	d	d	d	d	1	1
	**2012**	3380.	0.10	0.95	0.70	92.80	8.00	39.00	78.00	6.00	4.60	3.60	9.80	7.20	6.90	0.18	9.70
	**2013**	3500.	0.40	0.80	0.30	99.30	6.80	38.00	73.00	10.00	2.80	1.30	16.00	11.00	10.00	0.31	12.00
	**2014**	3600.	0.40	0.80	0.12	96.70	5.40	38.00	65.00	8.00	4.30	2.00	17.90	8.00	14.40	0.50	1.40
	**2015**	3500.	0.20	0.91	0.80	94.30	6.90	40.00	75.00	9.00	4.50	3.70	10.50	8.00	6.60	0.33	1.20
	**2016**	4100.	0.30	0.80	0.40	98.00	12.90	38.00	79.00	13.00	4.40	1.80	12.00	9.00	6.80	0.26	6.00
	**2017**	3900.	0.30	0.92	0.40	100.60	8.00	36.00	69.00	10.00	4.60	2.80	14.90	8.00	11.60	0.29	1.80
	**2018**	3700.	0.30	0.93	0.80	97.40	5.10	39.00	74.00	12.10	4.00	2.90	12.00	9.00	5.90	0.28	1.60
	**2019**	3700.	0.30	0.92	0.80	98.00	8.20	37.00	71.00	7.00	4.60	3.70	10.10	6.90	7.30	0.23	33.00
	**2020**	3600.	0.20	0.91	0.80	94.30	4.50	39.00	76.00	1.70	4.50	3.50	10.40	7.00	6.50	0.29	2.00
**Pointe Géologie**	**2021**	3900.	0.20	0.93	0.70	95.10	5.00	40.00	79.00	5.90	4.70	4.00	7.90	6.30	6.40	0.21	1.90
	**2018**	9000.	0.40	0.90	0.70	125.00	10.00	37.00	71.00	7.00	4.00	2.60	8.00	7.00	6.00	0.33	1600.0
	**2019**	9000.	0.40	0.92	0.70	115.00	9.10	38.00	76.00	7.00	4.30	3.10	8.00	6.50	5.70	0.34	190.00
**Atka Bay**	**2020**	9000	0.4	0.8	0.6	110.0	8.0	38.0	72.0	8.0	3.7	2.2	14.0	10.0	8.0	0.6	700.0

Supplementary Figure 2

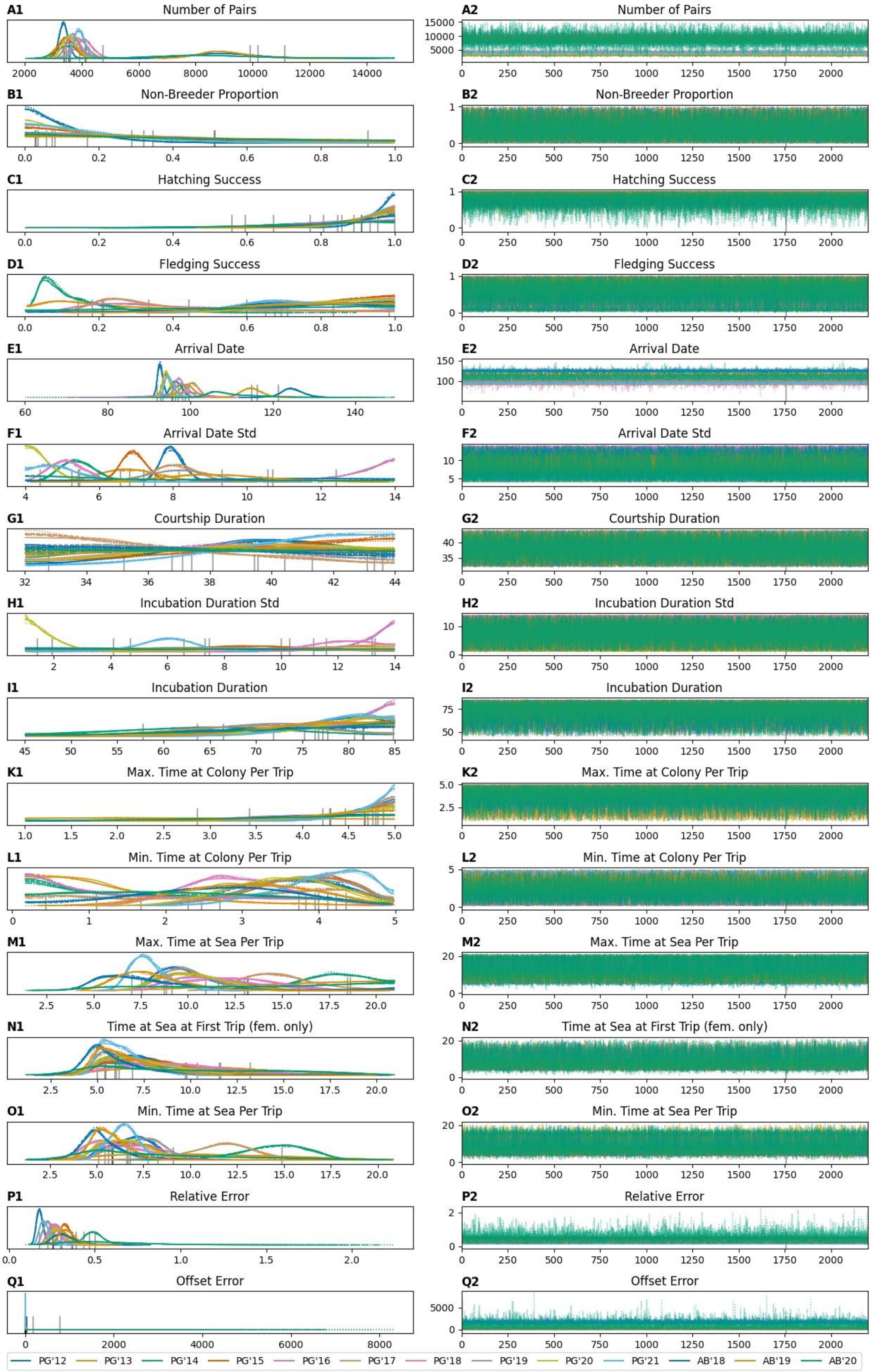

Sup. Fig. 2.: Plot of kernel density estimates and traces from the sampling process. Every row shows one parameter of the sampling process. The left column shows the kernel density estimates: On the X-axis the parameter value on the Y-axis the probability of that value. The right column shows the trace of the sampling process (after the removing the tuning samples): The X-axis shows the sample index from 0-2400. TheY-axis shows the parameter value associated with that sample. Different colors indicate different seasons. Different line styles indicate different chains (1 to 4) in the sampling process.

Supplementary Figure 3

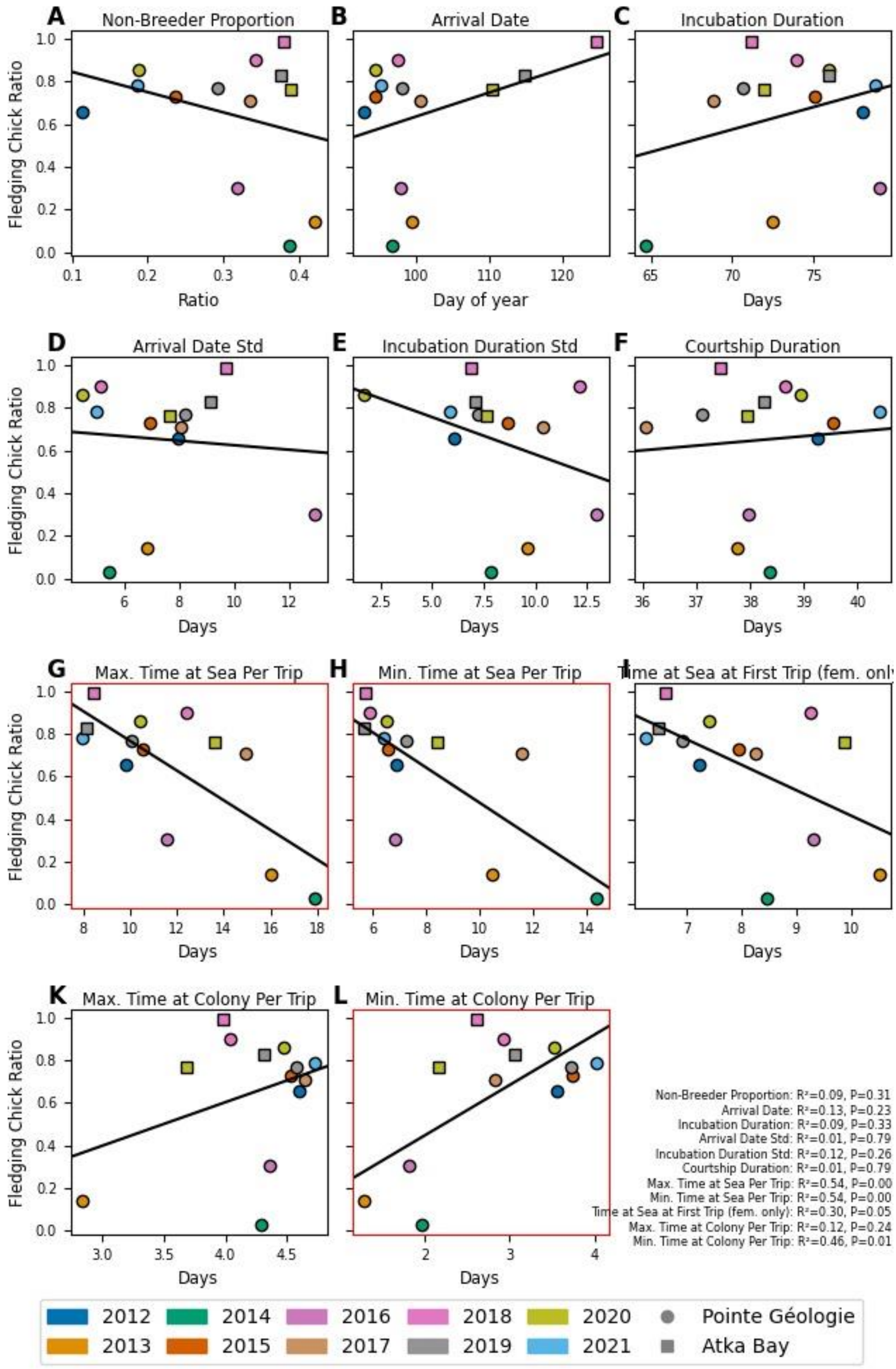

Sup. Fig. 3.: Correlation of model parameters with breeding success. The panels show the average predicted parameter value on X-axis and the ratio of fledging chicks to the number of breeders from manual observations on the Y-axis. Points show PG, squares show Atka Bay data. Colors indicate the year. The black line shows a linear regression fit performed on the data in the plot. We find significant correlation for the parameters c_{min}, s_{max}, and s_{min} indicated by a red outline of the plot box.

Supplementary Figure 4

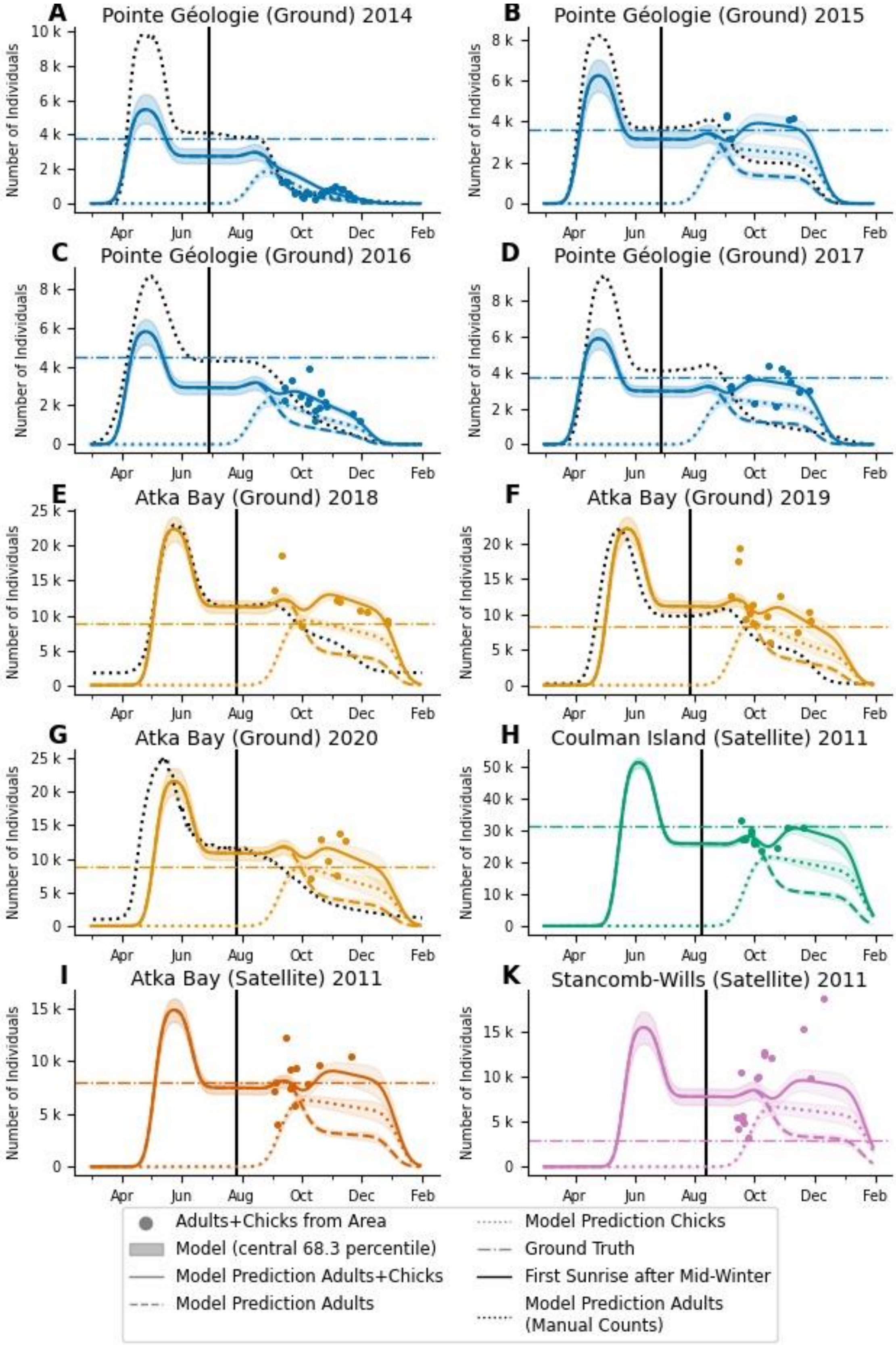

Sup. Fig. 4.: Model predictions for satellite-based data. The plots show the total number of individuals per breeding season and colony. The points show the total number of

individuals calculated by multiplying the colony covered area with the density predicted from meteorological values with the windchill model. Colony covered areas are measured either from ground (PG 2014-2017, AB 2018-2020) or from satellite (CI 2011, AB 2011, SW 2011) images. The colored dashed, dotted and solid lines represent the average number of adults, chicks and animals in total as predicted by the model based on the measured values represented in dots. The shaded areas indicate the 1-sigma interval (central 68.3 percentile). The dotted black line shows the number of adults predicted from a model fitted to manual count data including the whole season from March to January the following year (as in Fig. 2 C-F and Fig. 2 K-M). The horizontal dash-dotted lines show ground-truth values for the total number of breeding pairs from manual observations (PG, AB 2018-2020) or from [24] (CI 2011, AB 2011, SW 2011). The vertical solid line shows the first sunrise after mid winter at the respective colony locations. According to our findings for PG and AB we used this date to align the phenological patterns of the other colonies. Colors indicate the colony and data source (ground or satellite-based)

Supplementary Note 2

We calculate the R2 score as follows from two arrays x, and y containing the true and respective predicted values:

$u = \sum_i^N (x - y)^2$

$\mu = \sum_i^N x/N$

$v = \sum_i^N (x - \mu)^2$

$R2 = 1 - u / v$

We calculate the statistical significance of inter-colony variability as follows: We calculate the average parameter value for each season/colony combination. Then we pool the Atka Bay (AB) and Pointe Géologie (PG) values and perform a two-sided Mann-Whitney U statistics (using the python package scipy version 1.7.3). If the resulting p-value is smaller than 0.05, we accept the parameter as colony-variant.

We calculate the statistical significance of inter-year variability as follows: For each parameter set from the sampling chain, we calculate the pairwise differences of annual parameter values. This yields 45 distributions of parameter differences for the 10 years from Pointe Géologie and 3 distributions for the 3 years from Atka Bay. In these distributions zero equals no difference between years and a symmetric distribution around zero indicates no inter year variance. We count the number of samples left of zero and right of zero. We use Bonferroni-corrected significance values of 0.05/45 for Pointe Géologie and 0.05/3 for Atka Bay. If less than a significant portion of samples are on one side of zero, we accept the pair to be significantly different. If one of the inter year pairs for one colony is significantly different, we call the parameter inter-year variable at the respective colony.

We perform this test independently for Atka Bay and Pointe Géologie parameter sets.

Supplementary Note 3

The phenological model is a mechanistic description of the breeding behavior of emperor penguins using 14 parameters. The 14 parameters of the model are sampled with a Markov-Chain Monte-Carlo sampler. Their prior distributions are all uniform within fixed limits, as follows:

Arrival time:$t_0 \sim U$ (50d,150d)
Arrival time distribution width:$\Delta t_0 \sim U$ (4d,14d)
Duration of courtship:m ~ U (28d,42d)
Duration of female absence (incubation):b ~ U (50d,100d)
Female absence duration distribution width:$\Delta b \sim U$ (0d,14d)
Number of breeding pairs:BP ~ U (2000,15000)
Fraction of non-breeders:NB ~ U (0,1)
Hatching success:H ~ U (0,1)
Fledging success:F ~ U (0,1)
Maximal duration of stay at colony during foraging:$c_{max} \sim U$ (1d,5d)
Minimal duration of stay at colony during foraging:$c_{min} \sim U$ (1d,c_{max})
Maximal duration of foraging trip:$s_{max} \sim U$ (c_{max},21d)
Minimal duration of foraging trip:$s_{min} \sim U$ (c_{max},s_{max})
Duration of first foraging trip of females:$s_{fem} \sim U$ (c_{max},s_{max})

With those parameters, the model provides a function for the number of breeding males $N_M(t)$, breeding females $N_F(t)$, chicks $N_C(t)$, and non-breeders $N_{NB}(t)$ observed at the colony at a time t. These functions are based on presence/absence patterns of individuals, which again are based on a set of 26 events, when individuals can either enter or leave the colony. The numbers of individuals are therefore the sum of 26 presence functions $P_i(t)$ weighted by 26 participation factors f_i to account for the fact that not all animals participate in all events (see equations 2, 1, 3, and 4). We assume that each event timing is normally distributed with a mean t_i and a width Δt_i. The presence function $P_i(t)$, i.e. the fraction of individuals of a type (male breeder, female breeder, chick, or non-breeder) that have undergone an event and therefore entered or left the colony, is therefore an error function with the two parameters t_i and Δt_i: $P_i(t) = erf(\frac{t-t_i}{\Delta t_i})$.
The 26 x 4 participation factors f_i, 26 mean timings t_i,
and 26 distribution widths Δt_i are however not free parameters, but governed by knowledge about the species' breeding cycle and thereby reduced to the 14 original model parameters. In the following we define every factor, mean timing and distribution width and how it is calculated from the original parameters.

(1) $N_M(t) = BP \sum_{i=0}^{25} f_{M,i} P_i(t) = BP \sum_{i=0}^{25} f_{M,i} erf(\frac{t-t_i}{\Delta t_i})$

(2) $N_F(t) = BP \sum_{i=0}^{25} f_{F,i} P_i(t) = BP \sum_{i=0}^{25} f_{F,i} erf(\frac{t-t_i}{\Delta t_i})$

(3) $N_C(t) = BP \sum_{i=0}^{25} f_{C,i} P_i(t) = BP \sum_{i=0}^{25} f_{C,i} erf(\frac{t-t_i}{\Delta t_i})$

(4) $N_{NB}(t) = BP \sum_{i=0}^{25} f_{NB,i} P_i(t) = BP \sum_{i=0}^{25} f_{NB,i} erf(\frac{t-t_i}{\Delta t_i})$

The presence and absence of one individual would be described by a sharp step function (see Sup. Fig. 8A). However, as we are monitoring a whole colony, the step is likely ”blurred” by the individual variation in timing (see Sup. Fig. 8 B & C), resulting in a smooth sigmoidal function P_i, that describes the fraction of individuals that have already undergone the event. We assume that the distribution of individual event timing follows a Gaussian distribution with mean t_i and width Δt_i, leading to an error function shaped presence function ($P_i(t) = erf(\frac{t-t_i}{\Delta t_i})$)
(see Sup. Fig. 8D). The gaussian shape parameters t_i and Δt_i for each of the 26 events are calculated from the original 14 model parameters as follows.

- $t_0 = t_0$ is the arrival time.
- $t_1 = t_0+m$ is the first female departure, with m the duration of courtship.
- $t_2 = t_1 + b$ is the first female return and first male departure, with b the duration of female absence (incubation).
- $t_3 = t_2 + s_0$ is the first male return with s_0 the time spent at sea by the male breeders during their first foraging trip.
- $t_4 = t_3 + c_0$ is the second female departure with c_0 the time spent at the colony by both breeding partners.
- $t_5 = t_4 + s_1$
- $t_6 = t_5 + c_1$
- ...
- $t_{24} = t_{23} + c_{10}$
- $t_{25} = t_{24} + s_{11}$

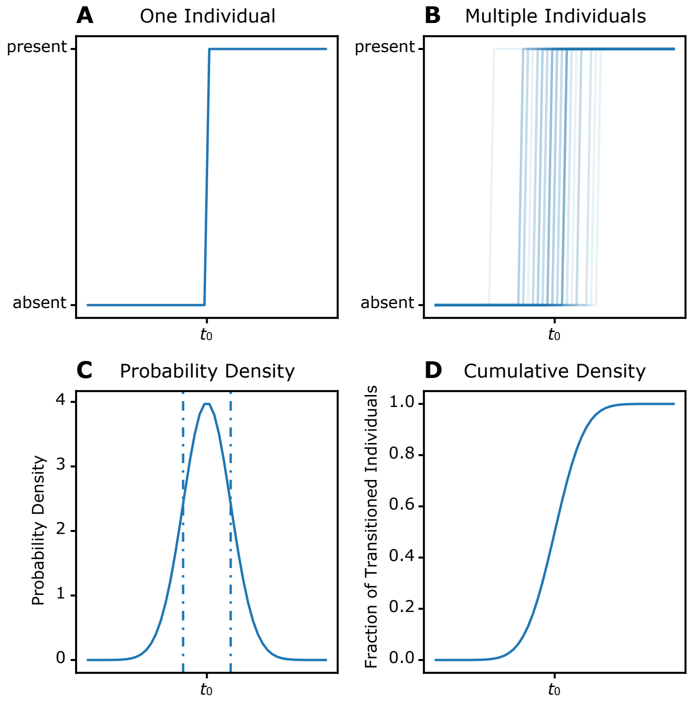

Sup. Fig 8: Presence/absence patterns for one ”entering” event at time t_i: A) The presence of one individual is a step response. B) Different individuals will enter at different times, creating a jitter on the presence/absence function. C) We assume that those different entering times follow a normal distribution with mean t_i and width Δt_i. D) The cumulative distribution function

of this normal distribution describes the fraction of individuals that has already entered the colony because of the event at t_i.

The time spent at sea s_i and at colony c_i during each foraging turnover is controlled by the model parameters s_{min},s_{max},c_{min}, and c_{max}, so that the time at sea and at colony perform a linear decrease throughout the breeding season.

(5) $s_i = s_{min} + \frac{11-i}{11} s_{max}\ for\ i \in [0,4,5,\ldots,11]$

(6) $c_i = c_{min} + \frac{10-i}{9} c_{max}\ for\ i \in [1,2,3,,\ldots,10]$

The special cases s_1,s_2,s_3, and c_0 arise from the first female departure being reportedly shorter than the male absence, and the first two times spent at colony being equally long:

$$s_1 = s_{fem}$$

$$s_2 = s_{fem}$$

$$s_3 = s_{min} + \frac{8}{11} s_{max} - s_{fem}$$

$$c_0 = c_{max}$$

Table 2 contains a recursive definition of every event timing. Table 3 contains an explicit formula for every event timing.

The distribution widths are governed by the two model parameters Δt_0 and
Δb, the width of arrival timing, and the width of the female return timing (see equation 7). We assume that only those two events are significantly impacted by external triggers (e.g. large storms) that desynchronize the behavior of individuals within a colony. We further assume, that a desynchronization in the beginning of the breeding season can not be recovered within one breeding season leading to a cumulative addition of the width parameters.

(7) $\Delta t_i = \Delta t_0\ if\ i \leq 1 \sqrt{\Delta t_0^2 + \Delta b^2}\ else$

As not all types of individuals (male breeders, female breeders, chicks, nonbreeders) and also not always all individuals of that type, but a fraction, participate in every event, the presence function P_i needs to be multiplied by a scaling factor $f_{X,i}$ with i ∈ [0,1,...,25] and X noting one of M,F,C, or NB for the individual type. These factors are shown in table 1 and follow the breeding pattern as known from literature. For example, the male breeders participate in arrival ($f_{M,0} = 1$), stay at the colony, while females leave at t_1 ($f_{M,1} = 0$). After the incubation phase (t_2) all males leave ($f_{M,2} = -1$) and so on. The factors do have to follow several mathematical constraints:

- $|f_{X,i}| \leq 1$ At max 100% of individuals of one type can participate in an event
- $\sum_{i=0}^{25} f_{X,i} = 0$ No individuals are at the colony after the end of the breeding season.
- $0 \leq \sum_{i=0}^{j} f_{X,i} \leq 1\ for\ j \in [0,1,\ldots,25]$ The number of present individuals at any time must lie between 0% and 100% of all individuals of that type.

These constraints are met by our definition of each factor as in table 1.

To estimate the free parameters of the model defined above, we use the Bayesian inference python package PYMC3 with the No-U-Turn Sampler (NUTS). The inference process takes the model structure and the measured data (number of observed adults $\hat{N}_j$ and the respective dates $\hat{t}_j$ for each observation j) as input, and outputs a series of random samples from the distribution of the inferred parameters. These (posterior) parameter distributions can then be

used to compute the mean inferred parameter value and to quantify the uncertainty tied to the parameter estimate.

To carry out the inference process, PYMC3 needs to know how likely the model could generate the observed data given certain parameter values. The probability of generating a data set given a model and its parameter values is called likelihood function. The likelihood is the probability density of the observation error distribution, but with the distribution parameters as variables (see equation 8).

We assume that the error of manually estimating the number of penguins is a relative error (if one counts 1,000 penguins and misses the true count by 10, they will likely miss by 100 on average if they count 10,000 penguins). Such a relative error is implemented by assuming that estimated penguin counts follow a log-normal distribution. The log-normal distribution has one additional shape parameter σ, which corresponds to the width of the distribution. The shape parameter encodes the size of estimation error that researchers make when counting penguins manually. Since we do not know the accuracy of the penguin count a-priori, the shape parameter represents an additional free parameter of the model and it is estimated from the data. As is common for such parameters in Bayesian inference, we choose a prior distribution that favors small values (Exponential Distribution), but still allows a wide range of estimation errors.

The total likelihood of all observations j is their product (see equation 9). Therefore the optimization of the log-normal distributed error function is essentially a minimization of the sum of squared differences of the log values $\sum_j \left(ln\ (\widehat{N_j})\ - ln\ (N(\widehat{t_j}))\right)^2$.

(8) $L\left(N(\widehat{t_j}), \sigma | \widehat{N_j}\right)\ =\ \frac{1}{\widehat{N_j}\sigma\sqrt{2\pi}}\, exp\left(-\frac{\left(ln(\widehat{N_j})\ -\ ln(N(\widehat{t_j}))\right)^2}{2\sigma^2}\right)$

(9) $\prod_j L\left(N(\widehat{t_j}), \sigma | \widehat{N_j}\right)\ = \frac{1}{\sigma\sqrt{2\pi}} \prod_j \frac{1}{\widehat{N_j}}\, exp\left(-\frac{\left(ln(\widehat{N_j})\ -\ ln(N(\widehat{t_j}))\right)^2}{2\sigma^2}\right)$

The ratio of participating individuals for each of the 26 events are defined as follows:

$f_{M,0} = 1$	$f_{F,0} = 1$	$f_{C,0} = 0$	$f_{NB,0} = NB$
$f_{M,1} = 0$	$f_{F,1} = -1$	$f_{C,1} = 0$	$f_{NB,1} = -NB$
$f_{M,2} = -1$	$f_{F,2} = 1$	$f_{C,2} = 0$	$f_{NB,2} = 0$
$f_{M,3} = 0$	$f_{F,3} = H - 1$	$f_{C,3} = 0$	$f_{NB,3} = 0$
$f_{M,4} = H$	$f_{F,4} = 0$	$f_{C,4} = 0$	$f_{NB,4} = 0$
$f_{M,5} = 0$	$f_{F,5} = -H$	$f_{C,5} = 0$	$f_{NB,5} = 0$
$f_{M,6} = 0$	$f_{F,6} = H$	$f_{C,6} = 0$	$f_{NB,6} = 0$
$f_{M,7} = -H$	$f_{F,7} = 0$	$f_{C,7} = 0$	$f_{NB,7} = 0$
$f_{M,8} = 0$	$f_{F,8} = -H$	$f_{C,8} = 0$	$f_{NB,8} = 0$
$f_{M,9} = 0$	$f_{F,9} = 0$	$f_{C,9} = 0$	$f_{NB,9} = 0$
$f_{M,10} = F^{\frac{1}{7}}$	$f_{F,10} = F^{\frac{1}{7}}$	$f_{C,10} = H - F^{\frac{1}{7}}$	$f_{NB,10} = 0$
$f_{M,11} = -F^{\frac{1}{7}}$	$f_{F,11} = -F^{\frac{1}{7}}$	$f_{C,11} = F^{\frac{2}{7}} - F^{\frac{1}{7}}$	$f_{NB,11} = 0$
$f_{M,12} = F^{\frac{2}{7}}$	$f_{F,12} = F^{\frac{2}{7}}$	$f_{C,12} = 0$	$f_{NB,12} = 0$
$f_{M,13} = -F^{\frac{2}{7}}$	$f_{F,13} = -F^{\frac{2}{7}}$	$f_{C,13} = F^{\frac{3}{7}} - F^{\frac{2}{7}}$	$f_{NB,13} = 0$
$f_{M,14} = F^{\frac{3}{7}}$	$f_{F,14} = F^{\frac{3}{7}}$	$f_{C,14} = 0$	$f_{NB,14} = 0$
$f_{M,15} = -F^{\frac{3}{7}}$	$f_{F,15} = -F^{\frac{3}{7}}$	$f_{C,15} = F^{\frac{4}{7}} - F^{\frac{3}{7}}$	$f_{NB,15} = 0$
$f_{M,16} = F^{\frac{4}{7}}$	$f_{F,16} = F^{\frac{4}{7}}$	$f_{C,16} = 0$	$f_{NB,16} = 0$
$f_{M,17} = -F^{\frac{4}{7}}$	$f_{F,17} = -F^{\frac{4}{7}}$	$f_{C,17} = F^{\frac{5}{7}} - F^{\frac{4}{7}}$	$f_{NB,17} = 0$
$f_{M,18} = F^{\frac{5}{7}}$	$f_{F,18} = F^{\frac{5}{7}}$	$f_{C,18} = 0$	$f_{NB,18} = 0$
$f_{M,19} = -F^{\frac{5}{7}}$	$f_{F,19} = -F^{\frac{5}{7}}$	$f_{C,19} = F^{\frac{6}{7}} - F^{\frac{5}{7}}$	$f_{NB,19} = 0$
$f_{M,20} = F^{\frac{6}{7}}$	$f_{F,20} = F^{\frac{6}{7}}$	$f_{C,20} = 0$	$f_{NB,20} = 0$
$f_{M,21} = -F^{\frac{6}{7}}$	$f_{F,21} = -F^{\frac{6}{7}}$	$f_{C,21} = F^{\frac{7}{7}} - F^{\frac{6}{7}}$	$f_{NB,21} = 0$
$f_{M,22} = F^{\frac{7}{7}}$	$f_{F,22} = F^{\frac{7}{7}}$	$f_{C,22} = 0$	$f_{NB,22} = 0$
$f_{M,23} = -F^{\frac{7}{7}}$	$f_{F,23} = -F^{\frac{7}{7}}$	$f_{C,23} = 0$	$f_{NB,23} = 0$
$f_{M,24} = 0$	$f_{F,24} = 0$	$f_{C,24} = 0$	$f_{NB,24} = 0$
$f_{M,25} = 0$	$f_{F,25} = 0$	$f_{C,25} = -F$	$f_{NB,25} = 0$

The event timing for each individual event is defined as follows:

$t_0 = t_0$
$t_1 = t_0 + m$
$t_2 = t_1 + b$

$t_3 = t_2 + s_0 = t_2 + s_{min} + \frac{11}{11} s_{max}$
$t_4 = t_3 + c_0 = t_3 + c_{min} + \frac{9}{9} c_{max}$
$t_5 = t_4 + s_1 = t_4 + s_{fem}$
$t_6 = t_5 + c_1 = t_5 + c_{min} + \frac{9}{9} c_{max}$
$t_7 = t_6 + s_2 = t_6 + s_{fem}$
$t_8 = t_7 + c_2 = t_7 + c_{min} + \frac{8}{9} c_{max}$
$t_9 = t_8 + s_3 = t_8 + s_{min} + \frac{8}{11} s_{max} - s_{fem}$
$t_{10} = t_9 + c_3 = t_9 + c_{min} + \frac{7}{9} c_{max}$
$t_{11} = t_{10} + s_4 = t_{10} + s_{min} + \frac{7}{11} s_{max}$
$t_{12} = t_{11} + c_4 = t_{11} + c_{min} + \frac{6}{9} c_{max}$
$t_{13} = t_{12} + s_5 = t_{12} + s_{min} + \frac{6}{11} s_{max}$
$t_{14} = t_{13} + c_5 = t_{13} + c_{min} + \frac{5}{9} c_{max}$
$t_{15} = t_{14} + s_6 = t_{14} + s_{min} + \frac{5}{11} s_{max}$
$t_{16} = t_{15} + c_6 = t_{15} + c_{min} + \frac{4}{9} c_{max}$
$t_{17} = t_{16} + s_7 = t_{16} + s_{min} + \frac{4}{11} s_{max}$
$t_{18} = t_{17} + c_7 = t_{17} + c_{min} + \frac{3}{9} c_{max}$
$t_{19} = t_{18} + s_8 = t_{18} + s_{min} + \frac{3}{11} s_{max}$
$t_{20} = t_{19} + c_8 = t_{19} + c_{min} + \frac{2}{9} c_{max}$
$t_{21} = t_{20} + s_9 = t_{20} + s_{min} + \frac{2}{11} s_{max}$
$t_{22} = t_{21} + c_9 = t_{21} + c_{min} + \frac{1}{9} c_{max}$
$t_{23} = t_{22} + s_{10} = t_{22} + s_{min} + \frac{1}{11} s_{max}$
$t_{24} = t_{23} + c_{10} = t_{23} + c_{min} + \frac{0}{9} c_{max}$
$t_{25} = t_{24} + s_{10} = t_{24} + s_{min} + \frac{0}{11} s_{max}$

Expanding these definitions leads to the following formulas for the timing of each event.

$t_0 = t_0$
$t_1 = t_0 + m$
$t_2 = t_0 + m + b$
$t_3 = t_0 + m + b + s_{min} + s_{max}$
$t_4 = t_0 + m + b + s_{min} + s_{max} + c_{min} + c_{max}$
$t_5 = t_0 + m + b + s_{min} + s_{max} + c_{min} + c_{max} + s_{fem}$
$t_6 = t_0 + m + b + s_{min} + s_{max} + 2\,c_{min} + 2\,c_{max} + s_{fem}$
$t_7 = t_0 + m + b + s_{min} + s_{max} + 2\,c_{min} + 2\,c_{max} + 2\,s_{fem}$
$t_8 = t_0 + m + b + s_{min} + s_{max} + 3\,c_{min} + \frac{26}{9}\,c_{max} + 2\,s_{fem}$
$t_9 = t_0 + m + b + 2\,s_{min} + \frac{19}{11}\,s_{max} + 3\,c_{min} + \frac{26}{9}\,c_{max} + s_{fem}$
$t_{10} = t_0 + m + b + 2\,s_{min} + \frac{19}{11}\,s_{max} + 4\,c_{min} + \frac{33}{9}\,c_{max} + s_{fem}$
$t_{11} = t_0 + m + b + 3\,s_{min} + \frac{26}{11}\,s_{max} + 4\,c_{min} + \frac{33}{9}\,c_{max} + s_{fem}$
$t_{12} = t_0 + m + b + 3\,s_{min} + \frac{26}{11}\,s_{max} + 5\,c_{min} + \frac{39}{9}\,c_{max} + s_{fem}$
$t_{13} = t_0 + m + b + 4\,s_{min} + \frac{32}{11}\,s_{max} + 5\,c_{min} + \frac{39}{9}\,c_{max} + s_{fem}$
$t_{14} = t_0 + m + b + 4\,s_{min} + \frac{32}{11}\,s_{max} + 6\,c_{min} + \frac{44}{9}\,c_{max} + s_{fem}$
$t_{15} = t_0 + m + b + 5\,s_{min} + \frac{37}{11}\,s_{max} + 6\,c_{min} + \frac{44}{9}\,c_{max} + s_{fem}$
$t_{16} = t_0 + m + b + 5\,s_{min} + \frac{37}{11}\,s_{max} + 7\,c_{min} + \frac{48}{9}\,c_{max} + s_{fem}$
$t_{17} = t_0 + m + b + 6\,s_{min} + \frac{41}{11}\,s_{max} + 7\,c_{min} + \frac{48}{9}\,c_{max} + s_{fem}$
$t_{18} = t_0 + m + b + 6\,s_{min} + \frac{41}{11}\,s_{max} + 8\,c_{min} + \frac{51}{9}\,c_{max} + s_{fem}$
$t_{19} = t_0 + m + b + 7\,s_{min} + \frac{44}{11}\,s_{max} + 8\,c_{min} + \frac{51}{9}\,c_{max} + s_{fem}$
$t_{20} = t_0 + m + b + 7\,s_{min} + \frac{44}{11}\,s_{max} + 9\,c_{min} + \frac{53}{9}\,c_{max} + s_{fem}$
$t_{21} = t_0 + m + b + 8\,s_{min} + \frac{46}{11}\,s_{max} + 9\,c_{min} + \frac{53}{9}\,c_{max} + s_{fem}$
$t_{22} = t_0 + m + b + 8\,s_{min} + \frac{46}{11}\,s_{max} + 10\,c_{min} + 6\,c_{max} + s_{fem}$
$t_{23} = t_0 + m + b + 9\,s_{min} + \frac{47}{11}\,s_{max} + 10\,c_{min} + 6\,c_{max} + s_{fem}$
$t_{24} = t_0 + m + b + 9\,s_{min} + \frac{47}{11}\,s_{max} + 11\,c_{min} + 6\,c_{max} + s_{fem}$
$t_{25} = t_0 + m + b + 10\,s_{min} + \frac{47}{11}\,s_{max} + 11\,c_{min} + 6\,c_{max} + s_{fem}$

Supplementary Table 2

The table contains the breeding phenology event observations and predictions. N.A. = “Not Available”

**Season**		**2012**	**2013**	**2014**	**2015**	**2016**	**2017**	**2018**	**2019**	**2020**	**2021**
**Arrival**	**true**	2012-03-26	2013-04-06	2014-03-31	2015-03-30	2016-03-31	2017-04-01	2018-04-12	2019-04-05	2020-03-31	2021-03-29
	**predicted**	2012-04-02	2013-04-10	2014-04-07	2015-04-05	2016-04-07	2017-04-11	2018-04-08	2019-04-08	2020-04-04	2021-04-06
**Female First Departure**	**true**	2012-05-02	2013-04-30	2014-05-05	2015-05-12	2016-04-29	2017-04-26	2018-05-05	2019-05-03	2020-05-05	2021-05-02
	**predicted**	2012-05-12	2013-05-18	2014-05-16	2015-05-14	2016-05-15	2017-05-17	2018-05-17	2019-05-16	2020-05-13	2021-05-16
**Female Return**	**true**	2012-07-05	2013-07-26	2014-07-31	2015-07-18	2016-07-19	2017-07-27	2018-07-29	2019-07-26	2020-07-19	2021-07-26
	**predicted**	2012-07-29	2013-07-29	2014-07-19	2015-07-28	2016-08-02	2017-07-25	2018-07-30	2019-07-25	2020-07-28	2021-08-03
**Male Return**	**true**	NA	2013-08-19	2014-08-11	2015-08-04	2016-08-01	2017-08-06	2018-08-08	2019-08-08	2020-08-06	2021-08-09
	**predicted**	2012-08-12	2013-08-17	2014-08-10	2015-08-12	2016-08-18	2017-08-14	2018-08-15	2019-08-09	2020-08-11	2021-08-15
**Emancipation**	**true**	2012-08-23	2013-08-19	2014-08-16	2015-08-20	2016-08-17	2017-08-19	2018-08-17	2019-08-15	2020-08-09	2021-08-16
	**predicted**	2012-08-28	2013-09-02	2014-08-27	2015-08-29	2016-09-05	2017-08-31	2018-09-01	2019-08-25	2020-08-28	2021-08-31
**Fledging**	**true**	2012-12-14	NA	2015-01-07	NA	2016-12-30	2017-12-27	2018-12-04	NA	2020-12-03	2021-12-02
	**predicted**	2012-12-06	2013-12-28	2015-01-25	2015-12-09	2016-12-08	2018-01-12	2018-12-07	2019-12-06	2020-12-05	2021-12-04

Supplementary Table 3

The table contains the numerical values for the best estimate of the windchill model parameters.

	cT	cW	cR	cH	Tc	sigma
**mean**	-0.023845	0.079986	-0.000767	0.008434	-4.903825	0.731349
**std**	0.003476	0.007152	0.000133	0.001784	0.909726	0.023509

Supplementary Table 4

The table contains the numerical values of the best estimate of the satellite based phenological model parameters.

colony	unit	Point Géologie (Ground)				Atka Bay (Ground)			Coulman Island (SAT)	Atka Bay (SAT)	Stancomb-Wills (SAT)
**Season**		**2014**	**2015**	**2016**	**2017**	**2018**	**2019**	**2020**	**2011**	**2011**	**2011**
**Number of Pairs**	1	2800	3200	2900	3000	11200	11000	10800	25600	7300	7700
**Number of Pairs (Year)** [24]	1	-	-	-	-	-	-	-	25298 (2009)	9657 (2009)	5455 (2009)
**Fledging Success**	1	0.09	0.80	0.37	0.80	0.70	0.50	0.60	0.80	0.80	0.80
**Relative Error**	1	0.62	0.23	0.25	0.25	0.19	0.27	0.27	0.09	0.27	0.46
**Offset Error**	1	10.00	11.00	10.00	10.00	10.00	10.00	10.00	10.00	10.00	10.00

Supplementary Note 4

We define “First Sunrise after mid winter” as follows: We used the python package astropy (https://www.astropy.org/, version 5.2.2) to calculate the sun’s elevation at noon throughout the year at the respective colony locations. We define the first sunrise after mid winter as the first day with a noon elevation over 0° after the winter solstice.

Supplementary Note 5
In satellite-based surveys, colony covered area is not converted to a total number of individuals on the day of image recording, but to a total number of breeders. For this, the studies employ a combined conversion factor that corrects for phenological and windchill effects. The conversion factor is close to 1 (e.g 0.93 in [24]) We compute a conversion factor *CF* from the number of breeding pairs from manual observations and the annual average of colony-covered area from ground based images as *CF = BP/A*. For the colonies and seasons in our study we find the following values:
CF = 5.98 (Pointe Géologie 2014), 0.90 (PG 2015), 2.13 (PG 2016), 1.14 (PG 2017), 0.78 (Atka Bay 2018), 0.80 (AB 2019), 0.82 (AB 2020).

Supplementary Note 6
We use the windchill model to predict the density for each time point, where we measured the colony area. We calculate the number of individuals by multiplying the area with the density. In the case of ground-based images, we manually select the colony area by drawing a polygon. For spread-out colonies, average densities below 1 animal / m2 are possible. In the case of satellite images, by contrast, the colony area is extracted based on a pixel-wise classification, with each pixel being classified as "penguin" or "not-penguin". As the pixels are approximately 1 m^2 in size, the minimum animal density associated with a pixel is therefore one animal/m^2. To account for this, we constrain the lower bound of the predicted density to 1 animal/m2.

Supplementary Figure 5

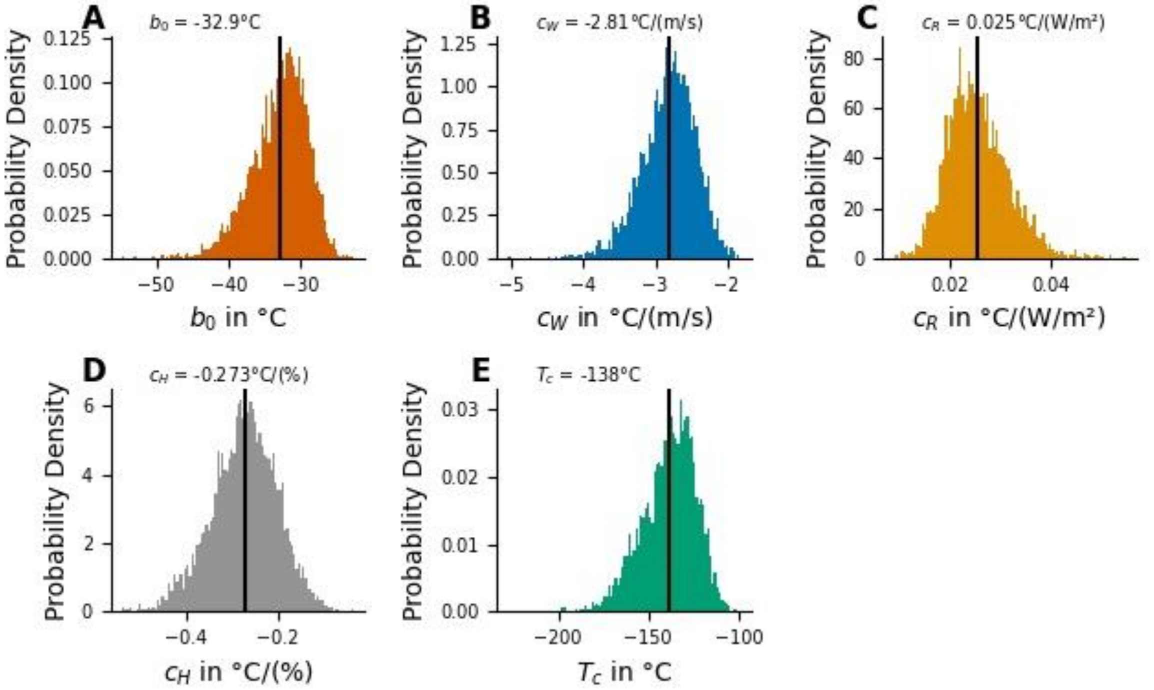

Sup. Fig. 5.: Parameter distribution of windchill model. The plots show the probability density for the 5 parameters of the windchill model b_0 (**A**), c_W (**B**), c_R (**C**), c_H (**D**), and T_C (**E**) as sampled by the windchill model. The solid vertical line and text in the figure show the

average value for each parameter.

Supplementary Figure 6

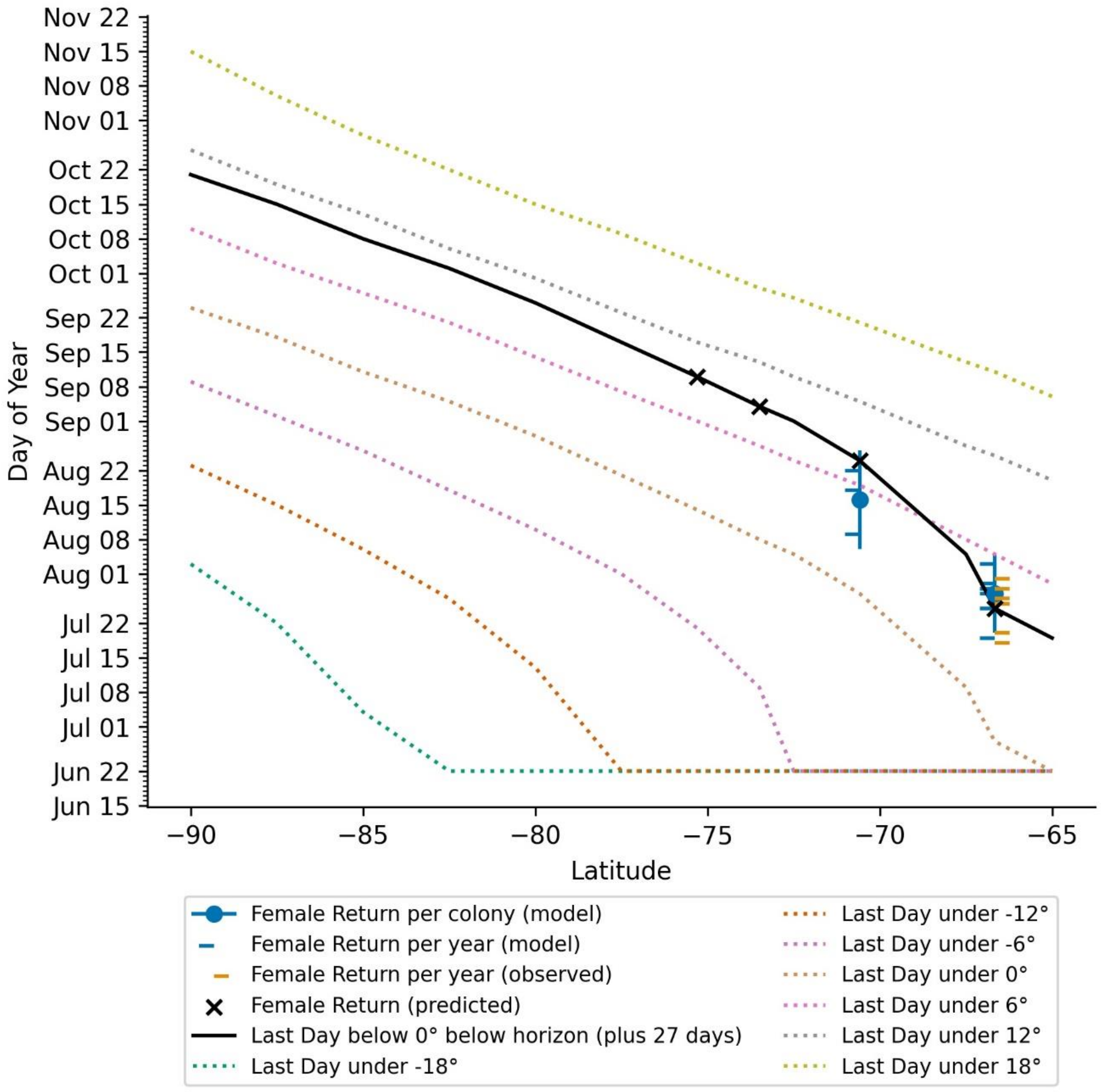

Sup. Fig. 6.: Day of first sunrise after midwinter for different latitudes. The plot shows latitude against the day of year. The blue dots and error bars show the average +/- standard deviation predicted day of female return as predicted by our phenology model. The blue left ticks show the predicted day of female return per season as predicted by the model. The orange right ticks show the day of female return as of manual observations. The dashed lines show the last day with a maximum solar elevation of -18°, -12°, -6°, 0°, 6°, 12°, and 18° at the respective latitude on the x-axis. Note: The lines for the low elevations (-18-0°) flatten out on June 21 for higher latitudes, because there are no days with such a small maximum sun elevation at those latitudes. The black line shows the day 27.4 days later than the last day with a maximum sun elevation smaller than 0°, which is our best fit for the prediction of the Female Return. The black crosses show the female return predicted for the latitudes of the 4

colonies in our study (Coulman Island, Stancomb-Wills, Atka Bay, and Pointe Géologie) according to this prediction.

Supplementary Table 5

Comparison of different censuses of Atka Bay (AB), Stancomb Wills (SW), Coulman Island (CI), and Pointe Géologie (PG)

**Location**	**Year**	**Type of Survey**	**Source**	**Number of Breeders**
CI	2006	Ground/Aerial	(*23*)	31432
CI	2009	Satellite	(*23*)	25298
CI	2011	Aerial	(*22*)	22687
CI	2011	Satellite	this study	25600
SW	1986	not documented	(*23*)	3000
SW	2009	Satellite	(*23*)	5455
SW	2011	Satellite	(*22*)	8814
SW	2011	Satellite	this study	7700
AB	1986	Ground/Aerial	(*23*)	8000
AB	2009	Satellite	(*23*)	9657
AB	2011	Satellite	(*22*)	7200
AB	2011	Satellite	this study	7300
AB	2018	Ground	this study	11200
AB	2019	Ground	this study	11000
AB	2020	Ground	this study	10800
PG	1987	Ground	(*23*)	2300
PG	2009	Satellite	(*23*)	2456
PG	2012	Ground	this study	3380
PG	2013	Ground	this study	3500
PG	2014	Ground	this study	3600
PG	2015	Ground	this study	3500
PG	2016	Ground	this study	4100
PG	2017	Ground	this study	3900
PG	2018	Ground	this study	3700
PG	2019	Ground	this study	3700

PG	2020	Ground	this study	3600
PG	2021	Ground	this study	3900

Supplementary Figure 7

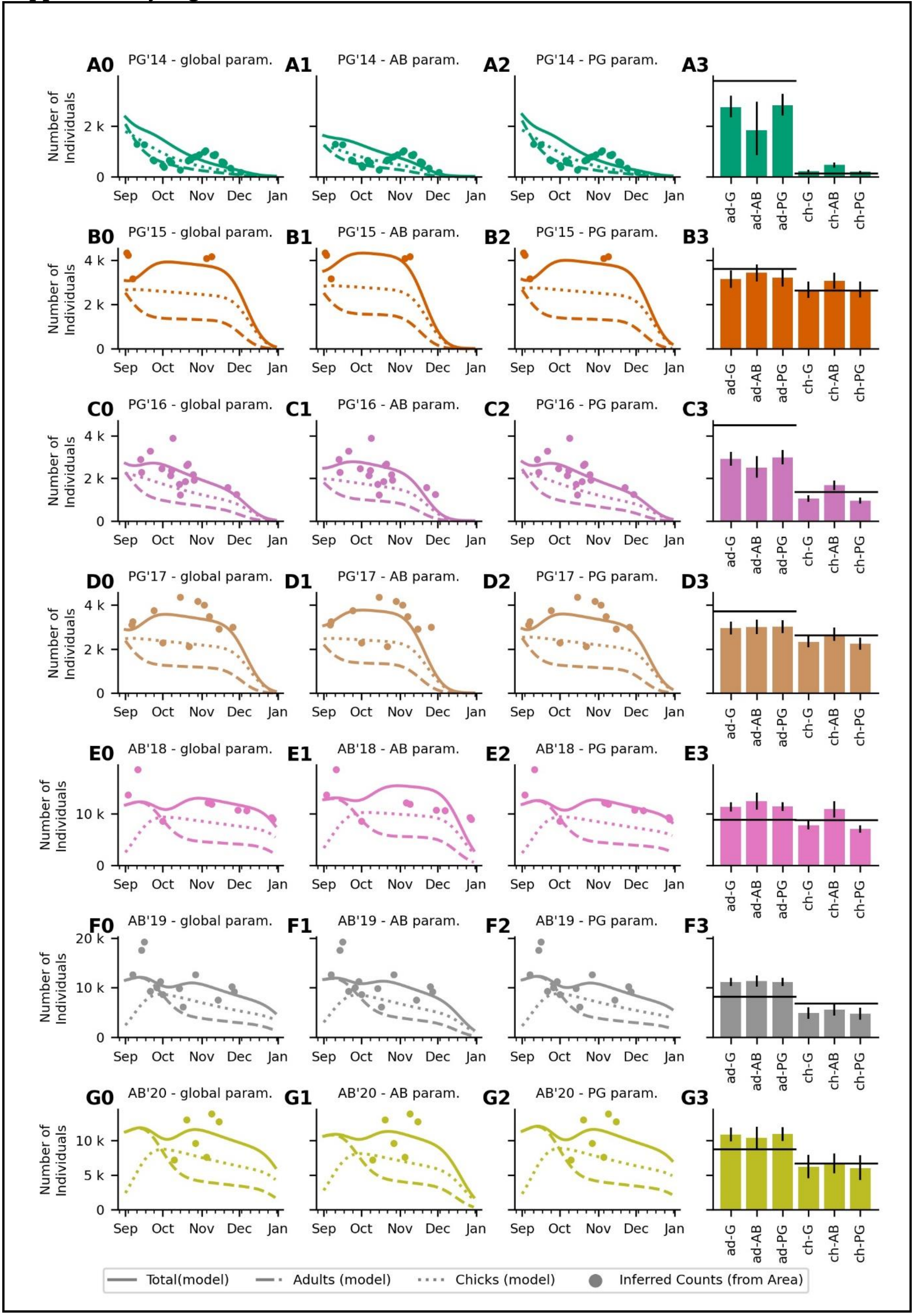

Sup. Fig. 7 Spatial cross correlation of the phenological model. The first three columns of panels (0-2) show results for the phenological model fitted with parameter values fixed to the average values from both colonies (0), Atka Bay (1), and Pointe Géologie (2). Points show the total number of individuals as predicted from colony covered areas by the windchill model. The lines show the phenological model prediction for the number of chick (dotted), adults (dashed), and total number of individuals (solid). The bar plots in the last column of panels (3) show the number adults (ad) and chicks (ch) predicted by the model based on average parameter values from both colonies (G = "global"), from Atka Bay (AB), and Pointe Géologie (PG). The error bars show the 1-sigma confidence interval. The black horizontal lines show the true observed number of individuals. The panel rows (A-G) show the results for different data points split by season and colony (Pointe Géologie 2014, 2015, 2016, 2017 (A-D), Atka Bay 2018, 2019, 2020 (E-G)).
