# Supplementary material for: Remote sensing of emperor penguin abundance and breeding success
Source: Nat Commun. 2024 May 29;15:4419. doi: 10.1038/s41467-024-48239-8 (PMC11137044; doi:10.1038/s41467-024-48239-8)
Supplement: Supplementary file 2 — Reporting Summary [file 41467_2024_48239_MOESM2_ESM.pdf]

nature portfolio

Corresponding author(s): Alexander Winterl, Daniel P. Zitterbart

Last updated by author(s): Apr 19, 2024

Reporting Summary

Nature Portfolio wishes to improve the reproducibility of the work that we publish. This form provides structure for consistency and transparency in reporting. For further information on Nature Portfolio policies, see our Editorial Policies and the Editorial Policy Checklist.

Statistics

For all statistical analyses, confirm that the following items are present in the figure legend, table legend, main text, or Methods section.

n/a	Confirmed	
☐	☒	The exact sample size (*n*) for each experimental group/condition, given as a discrete number and unit of measurement
☒	☐	A statement on whether measurements were taken from distinct samples or whether the same sample was measured repeatedly
☐	☒	The statistical test(s) used AND whether they are one- or two-sided<br>*Only common tests should be described solely by name; describe more complex techniques in the Methods section.*
☐	☒	A description of all covariates tested
☐	☒	A description of any assumptions or corrections, such as tests of normality and adjustment for multiple comparisons
☐	☒	A full description of the statistical parameters including central tendency (e.g. means) or other basic estimates (e.g. regression coefficient) AND variation (e.g. standard deviation) or associated estimates of uncertainty (e.g. confidence intervals)
☐	☒	For null hypothesis testing, the test statistic (e.g. *F*, *t*, *r*) with confidence intervals, effect sizes, degrees of freedom and *P* value noted<br>*Give P values as exact values whenever suitable.*
☐	☒	For Bayesian analysis, information on the choice of priors and Markov chain Monte Carlo settings
☐	☒	For hierarchical and complex designs, identification of the appropriate level for tests and full reporting of outcomes
☒	☐	Estimates of effect sizes (e.g. Cohen's *d*, Pearson's *r*), indicating how they were calculated

Our web collection on statistics for biologists contains articles on many of the points above.

Software and code

Policy information about availability of computer code

Data collection	We used camera observatories (SPOT, micrObs) and hand held cameras as described in the methods section. Publications describing the observatories are cited in the manuscript.
Data analysis	We used python and the packages pymc3, cameratransform, and clickpoints. Installation instructions, version numbers and custom code can be found in the github-repo (https://github.com/AlexanderWinterl/EmperorPenguinPhenology)

For manuscripts utilizing custom algorithms or software that are central to the research but not yet described in published literature, software must be made available to editors and reviewers. We strongly encourage code deposition in a community repository (e.g. GitHub). See the Nature Portfolio guidelines for submitting code & software for further information.

Data

Policy information about availability of data

All manuscripts must include a data availability statement. This statement should provide the following information, where applicable:

- Accession codes, unique identifiers, or web links for publicly available datasets
- A description of any restrictions on data availability
- For clinical datasets or third party data, please ensure that the statement adheres to our policy

The individual count data, area measurements from images and satellites, the meteorological measurements, the results of the bayesian sampling, the manually observed phenological event dates, and the manually observed breeding success measures are available at GitHub (https://github.com/whoi-mars/EmperorPenguinPhenology,) and provided in the Supplementary Information/Source data file. Source data are provided within this paper.

Research involving human participants, their data, or biological material

Policy information about studies with human participants or human data. See also policy information about sex, gender (identity/presentation), and sexual orientation and race, ethnicity and racism.

Reporting on sex and gender	not applicable.
Reporting on race, ethnicity, or other socially relevant groupings	not applicable.
Population characteristics	not applicable.
Recruitment	not applicable.
Ethics oversight	not applicable.

Note that full information on the approval of the study protocol must also be provided in the manuscript.

Field-specific reporting

Please select the one below that is the best fit for your research. If you are not sure, read the appropriate sections before making your selection.

☐ Life sciences ☐ Behavioural & social sciences ☒ Ecological, evolutionary & environmental sciences

For a reference copy of the document with all sections, see nature.com/documents/nr-reporting-summary-flat.pdf

Ecological, evolutionary & environmental sciences study design

All studies must disclose on these points even when the disclosure is negative.

Study description	We recorded images of whole Emperor Penguin colonies at two locations in Antarctica on a weekly to biweekly basis in 10 and 3 breeding seasons, counted the number of individuals once per image, and measured the area covered by the colony.
Research sample	The measurements represent the local population at their respective colony sites.
Sampling strategy	Does not apply, because we observed the whole colonies.
Data collection	The authors (AW, SR, AH, TB, MB, CC, DC, RC, CE, BF, AK, AM, DM, JM, SP, ES, CLB, DZ) participated in collecting the data.
Timing and spatial scale	Data was recorded every 7 days for 10 (PG, 2012-2021) and 3 (AB, 2018-2020) seasons. The different time frames arise from limitation of physical access to the colony. One season starts on 1st of March (PG) or 1st of April (AB) and ends on the same date of the next year. The shift in start and end date is due to the colonies difference in phenology. Individual missing dates or shifts in the schedule are due to limited access (usually heavy storms with no visibility). The data is provided together with the manuscript. Stating all individual missing points goes beyond the scope of this form.
Data exclusions	Images were excluded due to poor visibility.
Reproducibility	Our phenological model was applied to each breeding season at each colony independently. Reproducibility of the whole study is limited due to its duration (10 years).
Randomization	Does not apply.
Blinding	Our phenological model was applied to each breeding season at each colony independently.

Did the study involve field work? [x] Yes [ ] No

Field work, collection and transport

Field conditions	Meteorological conditions are part of the study data and can be found in the supplement in detail. The study was performed at two Emperor Penguin colonies in Antarcica. It was cold (-30°C to 0°C) and dry (no to little precipitation) at most occasions.
Location	Atka Bay (70° 40'S, 8° 16'W), Point Géologie (66° 40'S, 140° 01'E)
Access & import/export	Atka Bay: All procedures were approved by the German Environment Agency (Umweltbundesamt-UBA), permit no.: II 2.8–94033/100, II 2.8–94033/166, II 2.3 - 94032/1<br>Point Géologie: French ethics committee (APAFIS#29338-2020070210516365 and APAFIS#4897-2015110911016428) and the French Polar Environmental Committee of the Terres Australes et Antarctiques Françaises (TAAF project implementation and access permits 2012-117 & 2012-126, 2013-74 & 2013-82, 2014-116 & 2014-132, 2015-52 & 2015-105, 2016-76 & 2016-82, 2017-92 & 2017-102, 2018-116 & 2018-129, 2019-107 & 2019-115, 2020-65 & 2020-72, 2021-40 & 2021-51 & 2021-102)
Disturbance	We used automatically operated image and weather observatories. We minimized disturbance by reducing maintenance intervals, and keeping a distance of at least 50 m to the colony at any time during the observatory setup and operation. We adapted our SPOT-observatory design to minimize disturbance according to the guidelines of the German Environmental Agency.

Reporting for specific materials, systems and methods

We require information from authors about some types of materials, experimental systems and methods used in many studies. Here, indicate whether each material, system or method listed is relevant to your study. If you are not sure if a list item applies to your research, read the appropriate section before selecting a response.

Materials & experimental systems

n/a	Involved in the study	
[x]	[ ]	Antibodies
[x]	[ ]	Eukaryotic cell lines
[x]	[ ]	Palaeontology and archaeology
[ ]	[x]	Animals and other organisms
[x]	[ ]	Clinical data
[x]	[ ]	Dual use research of concern
[x]	[ ]	Plants

Methods

n/a	Involved in the study	
[x]	[ ]	ChIP-seq
[x]	[ ]	Flow cytometry
[x]	[ ]	MRI-based neuroimaging

Animals and other research organisms

Policy information about studies involving animals; ARRIVE guidelines recommended for reporting animal research, and Sex and Gender in Research

Laboratory animals	Does not apply.
Wild animals	Two whole Emperor Penguin colonies were images from a distance. No animals were captured or interacted with at any point.
Reporting on sex	Does not apply.
Field-collected samples	Image data was stored on hard drives.
Ethics oversight	German (Umweltbundesamt, UBA) and French (Polar Environmental Committee of the Terres Australes et Antarctiques Françaises, TAAF) Environmental agencies.

Note that full information on the approval of the study protocol must also be provided in the manuscript.
