# Supplementary material for: Remote sensing of emperor penguin abundance and breeding success
Source: Nat Commun. 2024 May 29;15:4419. doi: 10.1038/s41467-024-48239-8 (PMC11137044; doi:10.1038/s41467-024-48239-8)
Supplement: Supplementary file 4 — Description of Additional Supplementary Files [file 41467_2024_48239_MOESM4_ESM.pdf]

Title: Supplementary Data 1
Description: The file contains the manually conducted individual counts from ground based panoramic images. The file is machine readable and to be used with the software provided in the github repository (https://github.com/whoi-mars/EmperorPenguinPhenology).

Title: Supplementary Data 2
Description: The file contains manually conducted individual counts and area measurements from ground based images. In addition it contains the meteorological data recorded at the respective colony locations. The column headings are as follows: colony_name, ts (timestamp), Count (Number of adult penguins), Count Chicks (Number of chicks), A (Area in m^2 measured from images), temp_airK (air temperature in Kelvin), met_ff10 (windspeed at 10m height in m/s), met_rad (Solar radiation in W/m^2), met_humc (relative humidity corrected for measurements over ice). The file is machine readable and to be used with the software provided in the github repository (https://github.com/whoi-mars/EmperorPenguinPhenology).

Title: Supplementary Data 3
Description: The file contains manually conducted individual counts and area measurements from ground based images and satellite images. The satellite based measurements are for the colonies and seasons as follows: Atka Bay 2011, Coulman Island 2011, Stancomb Wills 2011. In addition it contains the meteorological data recorded at the respective colony locations or from ERA5 model, where satellite images were used. The column headings are as follows: colony_name, ts (timestamp), Count (Number of adult penguins), Count Chicks (Number of chicks), A (Area in m^2 measured from images), temp_airK (air temperature in Kelvin), met_ff10 (windspeed at 10m height in m/s), met_rad (Solar radiation in W/m^2), met_humc (relative humidity corrected for measurements over ice). The file is machine readable and to be used with the software provided in the github repository (https://github.com/whoi-mars/EmperorPenguinPhenology).

Title: Supplementary Data 4
Description: The file contains manually observed and model predicted number of breeders, lost eggs, dead chicks and fledged chicks for Atka Bay (2018-2020) and Pointe Géologie (2012-2021). The model prediction are best estimates from the Monte Carlo sampling chain.The file is machine readable and to be used with the software provided in the github repository (https://github.com/whoi-mars/EmperorPenguinPhenology).
